# Scoring and psychometric validation of the ‘Determinants of Intentions to Vaccinate’ (DIVA^©^) questionnaire

**DOI:** 10.1186/s12875-016-0539-3

**Published:** 2016-10-10

**Authors:** Luc Martinez, Fatoumata Fofana, François Raineri, Pascale Arnould, Khadra Benmedjahed, Guillaume Coindard, François Denis, Didier Duhot, Jean-Luc Gallais, Didier Seyler, Béatrice Tugaut, Benoit Arnould

**Affiliations:** 1 Mapi, Patient-Centered Outcomes, 27, rue de la Villette, 69003 Lyon, France; 2 French Society of General Medicine, Issy-les-Moulineaux, France; 3 Department of General Practice, University Paris-Sud, Le Kremlin-Bicêtre, France; 4 Department of bacteriology and virology, University of Limoges, Limoges, France; 5 Department of General Medicine, University Pierre-et-Marie-Curie, Paris, France; 6 Specialist in general medicine, International vaccination centre (2007–2015), Marseille, France; 7 Department of General Medicine, SMBH University of Paris 13, Bobigny, France

**Keywords:** Vaccination, Primary care physician, Attitude, Behavior, Public health, Psychometric validation

## Abstract

**Background:**

Primary care physicians (PCPs) play a key role regarding vaccination in France. The aims of the present study were to define the scoring rules and to assess the measurement properties of the ‘Determinants of Intentions to Vaccinate’ (DIVA^©^) questionnaire that aims to assess PCPs’ attitudes and beliefs toward vaccination.

**Methods:**

The DIVA questionnaire was derived from a literature review and PCPs focus groups. Scoring and early validation of the DIVA questionnaire were determined during a cross-sectional study conducted in France. During the study, PCPs had to complete the DIVA questionnaire for any of the six vaccine-preventable diseases (VPDs) to which they were randomly assigned (measles, pertussis, pneumococcus infection, seasonal influenza, human papillomavirus -HPV- infection and tetanus). Descriptive analyses of items and the analysis of the grouping of items into domains were conducted. Internal consistency reliability and construct validity was assessed according to each VPD.

**Results:**

The DIVA questionnaire was completed by 1,069 PCPs and was well accepted. The ‘Commitment of the PCP to the vaccination approach’ score showed very good internal consistency reliability (Cronbach’s alpha >0.70 overall and for each VPD). The construct validity of the DIVA questionnaire was confirmed.

**Conclusions:**

The DIVA questionnaire is a valid and reliable measure of PCPs’ attitudes and beliefs toward vaccination.

**Electronic supplementary material:**

The online version of this article (doi:10.1186/s12875-016-0539-3) contains supplementary material, which is available to authorized users.

## Background

Vaccination is the most effective medical intervention ever introduced [1, 2]. A recent study from the US Centers for Disease Control and Prevention reported that vaccination would have prevented 322 million illnesses and 732,000 premature deaths in children born during the period 1994–2013 [1, 3]. The smallpox vaccination has been responsible for the total eradication of the disease since its implementation in the 20th century [2]. Similarly, vaccines prevent millions of cases of infectious diseases worldwide, allowing for an increase in life expectancy from 59 to 70 years from 1970 through 2010 [2]. Nevertheless, vaccination coverage is lower than desired. From 2000 to 2010, the percentage of French respondents who declared they were unfavourable towards vaccination increased from 9 to 38 %, possibly due to the 2009 influenza A (H1N1) pandemic [4].

Vaccination experts acknowledge that public confidence in vaccination is waning [5, 6]. This “erosion in trust” is illustrated by the decline of vaccination coverage and is observed in most developed countries. Other evidence of the vaccine confidence gap is the increase in controversies surrounding various vaccines: the measles, mumps and rubella (MMR) vaccine in the United Kingdom, the hepatitis B vaccine in France and the H1N1 vaccine in many countries in 2009–2010 [5].

The relevance of the vaccination is also questioned by healthcare professionals. A study reported that French primary care physicians (PCPs) and paediatricians applied loosely the 2010 recommended vaccination schedule [7]. The tendency of clinicians reconsidering vaccination is not limited to France. One study showed that only 42 % of Italian paediatricians knew about the recommended vaccination schedule, and that only 10 % of Italian clinicians had positive opinions regarding the usefulness of recommended vaccinations [8]. In Canada, 37 % of healthcare professionals considered that there were too many vaccines and 36 % of them considered that a good lifestyle could eliminate the need for vaccination [9].

PCPs play a key role in the vaccination system in France. The relationship between the patient and the PCP and more generally healthcare professionals is the cornerstone of maintaining public confidence in vaccination, including addressing parents’ concerns on vaccines [9–11]. Several studies have shown PCPs’ influences in the recommendations on vaccination to their patients [12–14].

It is therefore crucial to understand the factors that influence PCPs’ commitment to vaccination. To address this need, the French Society of General Medicine (‘Société Française de Médecine Générale’ -SFMG-) developed a tool, the Determinants of Intentions to Vaccinate (DIVA^©^) questionnaire, following a rigorous qualitative stepwise methodology [15, 16]. The aims of the present study were to define the scoring rules and to assess the measurement properties of the DIVA tool.

## Materials and methods

### The DIVA questionnaire

The hypothetical conceptual framework of the DIVA questionnaire included 56 items, grouped into six thematic domains and one commitment domain: ‘Disease characteristics and expected benefits’ (9 items), ‘Properties of the vaccine’ (10 items), ‘Information about the vaccination’ (8 items), ‘Practical and organisational aspects’ (6 items), ‘Adaptation to the patient’s profile’ (11 items), ‘PCP’s individual experience’ (5 items) and ‘PCP’s commitment to the vaccination approach’ (7 items).

### Study setting and design

An observational, cross-sectional and multi-centre study involving French PCPs was conducted. All PCPs from the mailing list of the SFMG were invited by email to participate in the study. PCPs who agreed to enter the study were asked first to complete the socio-demographic form on a website. PCPs were then randomly assigned to one of the six vaccine-prevented diseases (VPDs) (measles, pertussis, pneumococcus infection, seasonal influenza, human papillomavirus -HPV- infection and tetanus) and were asked to complete the DIVA questionnaire. As tetanus is a compulsory vaccine in France and was very well accepted by PCPs during the qualitative phase, the tetanus disease group was considered as a control group in the study and thus included by design the smallest number of PCPs (the allocation ratio was one PCP for tetanus for three PCPs for each of the five other VPDs). There was no eligibility criterion to enter the study.

A modified Delphi panel, composed of the members of the DIVA scientific committee who are PCPs and specialists in vaccination, was carried out using the RAND/UCLA method in the early stage of the study [17]. It was conducted to appraise the position of the panellists on the six thematic domains of the DIVA questionnaire according to the six VPDs. The Delphi Panel was carried out into 3 phases. In the first phase, panellists were asked to complete a questionnaire with six propositions corresponding to the six thematic domains of the DIVA questionnaire for each VPD. Each proposition was presented in a numerical scale graduated from 1 to 9, with 1 indicating that the thematic domain was not a determinant of decision-making in vaccinating against the concerned VPD and 9 indicating that the thematic domain was a determinant of decision-making in vaccinating against the concerned VPD. In the second phase, panellists discussed online their responses. In the third phase, panellists were asked to complete the questionnaire again. At the end, each proposition was ranked according to the agreement between the panellists and the strength of the agreement.

### Statistical analyses

Item selection and definition of the scoring rule were based on the quality of completion of the DIVA questionnaire and on the distribution of item responses.

The DIVA questionnaire included both psychometric and composite domains. A psychometric domain is composed of items correlated with each other and measuring a single concept. In the DIVA questionnaire, the psychometric domain was the ‘PCP’s commitment to the vaccination approach’ domain. A composite domain is composed of items that are a combination of indicators of a common concept but not necessarily correlated with each other. In the DIVA questionnaire, composite domains were the six thematic domains. The statistical methods that are described below were adapted to the different natures of the concepts measured.

Principal component analysis (PCA) was performed to challenge the hypothetical conceptual framework of the DIVA questionnaire. This analysis aims at defining the grouping of items into domains by examining the self-organization of items [18]. Interpretable components with an eigenvalue greater than one represented the empirical domains.

An extension of the Rasch model, the Rating Scale Model (RSM) used for polytomic response scale [19], was performed on all items of the ‘PCP’s commitment to the vaccination approach’ domain to validate the existence of a unique underlying construct (called the “latent trait”) measuring the PCP’s commitment to vaccination [20]. All items were scaled with four categories (“Totally disagree”, “Somewhat disagree”, “Somewhat agree” and “Totally agree”). The Rasch model utilizes person and item parameters to determine the probability of an item score [21]. Raw scores for both persons and items are transformed into measures (known as “locations”) using a logistic mathematical function [21]. Model fit statistics were computed to indicate whether all items met the unidimensional measurement criterion using the residuals (range of acceptability from −2.5 to 2.5 [21]) and the item location to look at the deviation of the observed data from the model expectation [22]. The person-item threshold map, which is a graphical representation of person and item locations (measured in logits) plotted on the same latent trait along the x axis was also used.

The measurement properties of the DIVA questionnaire were assessed, including reliability and validity.

Reliability is the degree to which the instrument is free from measurement error. Internal consistency reliability (the extent to which items within a domain are consistent with each other and measure a single underlying concept) was assessed for the ‘PCP’s commitment to the vaccination approach’ domain, using Cronbach’s alpha [23].

Validity is the degree to which the instrument measures what it is supposed to measure. Construct validity was conducted to assess the degree to which DIVA thematic domains can distinguish the determinants of intentions to vaccinate among the six VPDs groups. The hypotheses for this analysis were formulated by the Delphi panel. The ranks of DIVA thematic domains for each VPD calculated from the Delphi panel were compared to those obtained from descriptive analyses.

### Ethical consideration

The present study was conducted in compliance with national ethical principles and the list of the members of the SFMG was submitted to the French National Commission for Data Processing and Privacy (‘Commission Nationale de l'Informatique et des Libertés’ -CNIL-).

## Results

### Population disposition

Among the 1,267 PCPs who completed the socio-demographic form, 1,069 PCPs completed at least one item of the DIVA questionnaire and constituted the analysis sample. The 1,069 PCPs were randomly assigned into the measles disease group (*N* = 214), the pertussis disease group (*N* = 203), the pneumococcus infection disease group (*N* = 196), the seasonal influenza disease group (*N* = 199), the HPV infection disease group (*N* = 184) and the tetanus disease group (*N* = 73).

### Characterization of PCPs

PCPs’ socio-demographic characteristics are presented in Table 1. The majority of PCPs were male (58 %) with an average age of 50 years. More than 90 % of PCPs had less than 40 % of paediatric activity. Around half of PCPs received medical representative visits and around a quarter had attended training on vaccination in the last 12 months before the beginning of the study. Eighty five percent of PCPs read the most recent weekly epidemiological record.

**Table 1:** Socio-demographic characteristics of PCPs (*N* = 1,069)

Variables	Analysis sample (*N* = 1069)
Age (years)	
n (missing)	1069 (0)
Mean (SD)	49.8 (11.6)
Min - Max	28.0–93.0
Gender (%)	
Male	58
Female	42
Level of pediatric activity (%)	
Between 0 and 20 %	46
Between 20 and 40 %	48
Between 40 and 60 %	5
More than 60 %	1
Medical representative visits (%)	52
Training on vaccination in the last 12 months (%)	23
Reading of the weekly epidemiological record of April 2013 (%)	85

*SD*, Standard deviation

### Scoring rules of the DIVA questionnaire

#### Quality of completion

The quality of completion of the questionnaire was very good. On average, there was less than one missing item per questionnaire. Ninety percent of all PCPs (*N* = 959) returned the DIVA questionnaire with all items completed. Forty three items out of 56 had less than one percent missing data and the 13 remaining items had between one and three percent missing data. The item with the highest number of missing data (*N* = 27) was about the PCP’s experience of the disease on a personal level.

#### Distribution of the responses to items

The distribution of the responses to items was good overall without bimodal distributions or floor effects. However, 21 items had a response choice used by more than 50 % of PCPs. Two pairs of redundant items were identified. The first pair concerned items ‘*efficacy of vaccination compared to other existing means of preventing’* and ‘*efficacy of vaccination compared to that of the range of curative treatments’*. The percentage of identical responses was 72 % and the Spearman correlation coefficient was 0.67. The redundancy might come from the similarity in the wording of these two items despite a clear difference in the concept (prevention vs. curative). Thus, the two items were differentiated by putting ‘prevention’ and ‘curative’ in bold letters. The second pair of redundant items concerned items ‘*I think about vaccination’* and ‘*I raise the subject of vaccination’*. The percentage of identical responses was 88 % and the Spearman correlation coefficient was 0.83. The redundancy might come from the strong link between reported intention (‘*I think about*’*)* and reported action (‘*I raise the subject of*’). To overcome the redundancy, the item ‘*I think about vaccination’* was removed from the questionnaire and the item ‘*I raise the subject of vaccination’* was kept as it was conceptually more consistent with the other items of the ‘PCP’s commitment to the vaccination approach’ domain, all depicting action rather than intention.

#### Final conceptual framework

The results of the PCA suggested an alternative conceptual framework of the DIVA questionnaire, including 10 empirical thematic domains and the ‘PCP’s commitment to the vaccination approach’ domain. However, the empirical conceptual framework did not provide significantly better results over the hypothetical conceptual framework. Besides, the empirical conceptual framework held more thematic domains than the hypothetical one (10 vs. 6 thematic domains respectively) and thus depicted a more complex picture of the determinants of intentions of PCPs to vaccinate. Therefore, as no loss of information was incurred by selecting a more parsimonious conceptual framework, the hypothetical conceptual framework with six thematic domains and one commitment domain was selected as the final structure of the DIVA questionnaire (see Additional file 1: Appendix A).

#### Rasch modelling

The Rasch model fit is presented in Table 2. The residuals were within the range of acceptability for all items except for items ‘*I am used to prescribing vaccination*’ (−6.14) and ‘*I insist on vaccination if the patient is reluctant’* (-2.73). The former asks about habits of vaccination and is less factual than other items. It was therefore decided to change the wording to ‘*I prescribe vaccinatio*n’ to be consistent with other items. The latter was deemed borderline and was kept unchanged. The order of items according to their location on the latent trait followed a theoretical model of the PCP’s commitment process and actions with vaccination. It ranged from −0.93 for the item ‘*My attitude toward prescribing the vaccine is in agreement with my beliefs’* to 0.88 for the item ‘*I make sure that my prescription for vaccination has been properly followed*’. The item ‘*Vaccination is a subject that interests me*’ was neutral in this process with its central position.

**Table 2:** Rasch individual item fit - Residuals and location of all items of the ‘PCP’s commitment to the vaccination approach’ domain (*N* = 1,069)

Item labels	Residuals	Location
My attitude toward prescribing the vaccine is in agreement with my beliefs	0.06	−0.93
I raise the subject of vaccination	−0.48	−0.54
I am used to prescribing vaccination	−6.14	−0.07
Vaccination is a subject that interests me	1.27	0.10
I insist on vaccination if the patient is reluctant	−2.73	0.56
I make sure that my prescription for vaccination has been properly followed	1.30	0.88

**Fig. 1:**
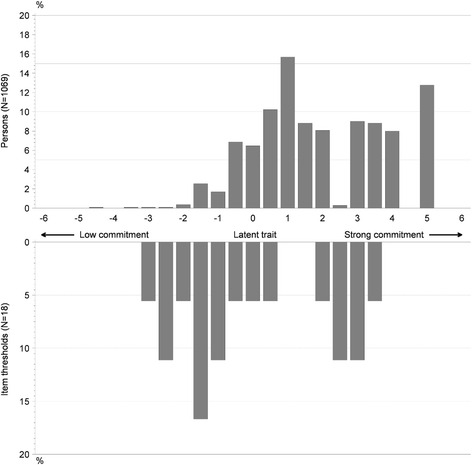
Person-item threshold distribution map of items from the ‘PCP’s commitment to the vaccination approach’ domain with items scaled with four categories (*N* = 1,069)

In the person-item threshold distribution map, the upper panel displays the frequency of recruited PCPs in the study according to their position on the latent trait, representing their level of commitment; the lower panel displays the “thresholds” (i.e. the point on the latent trait for which two adjacent response categories are equally probable) for the six DIVA items of the ‘PCP’s commitment to the vaccination approach’ domain (Fig. 1). The graph showed that all items were able to distinguish PCPs according to their level of commitment (items cover the latent trait from −4 to 4), and that PCPs who participated in the study were fairly committed to vaccination. Precisely, the graph shows that the locations corresponding to very strong commitment (at the extreme right end of the x-axis) and moderate commitment (in the middle of the x-axis) were partly covered by DIVA items whereas low commitment (at the extreme left end of the x-axis) was accurately covered.

#### Definition of the scoring rule

For all DIVA domains, the score was obtained by calculating the mean of non-missing items and applying a linear transformation resulting in a score ranging from 0 to 100. For the six thematic domains, a higher score is associated with a better perception of vaccination. For the commitment domain, a higher score is associated with stronger commitment toward vaccination. All scores were calculated if at least half the items within a domain were completed.

### Ability of DIVA scores to detect difference

**Fig. 2:**
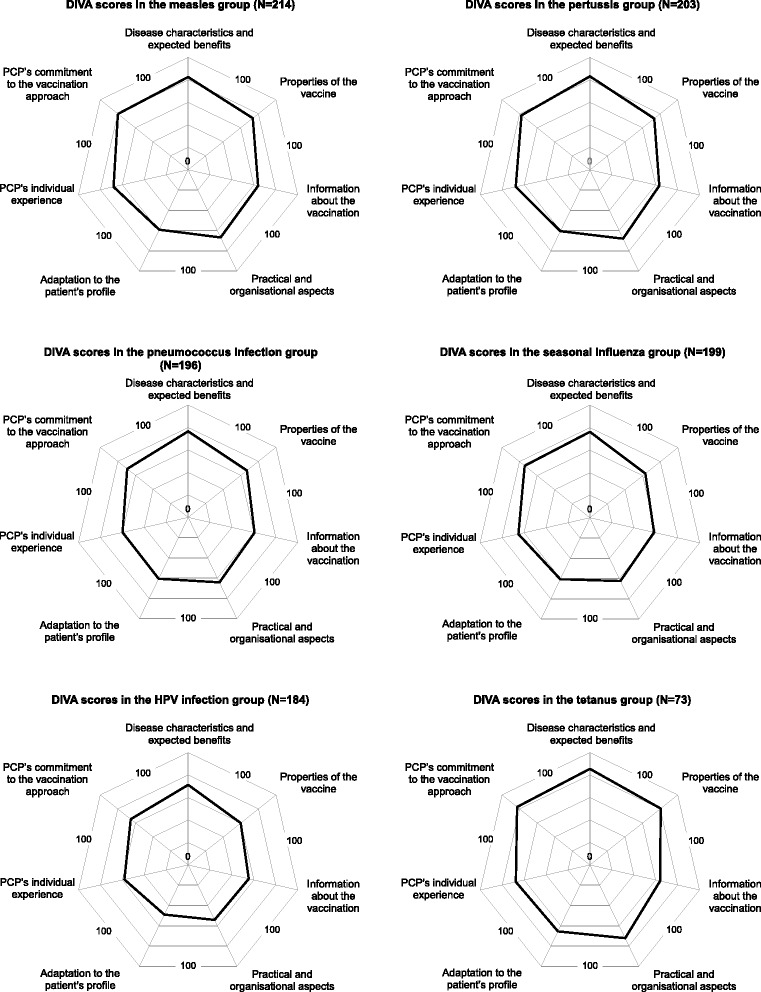
Score distribution of the DIVA questionnaire in the six vaccine-preventable diseases (*N* = 1,069)

The distribution of DIVA scores across the six VPDs is detailed in Fig. 2. Overall, the ‘Adaptation to the patient’s profile’, the ‘Information about the vaccination’ and the ‘Practical and organisational aspect’ domains showed the lowest mean scores (58.4, 60.7, 63.9 respectively). The mean scores were the lowest in the pneumococcus infection and the HPV infection diseases. On the contrary, the ‘Disease characteristics and expected benefits’ and the ‘PCP’s commitment to the vaccination approach’ domains showed the highest mean scores overall (78.7 and 74.2 respectively). The mean scores were at the highest for the measles, the pertussis and the tetanus diseases.

### Measurement properties

Cronbach’s alpha coefficients of the ‘PCP’s commitment to the vaccination approach’ score are presented in Table 3. They were above the recommended threshold of 0.70 for all diseases, ranging from 0.80 (for tetanus) to 0.87 (for measles and pneumococcus infection).

**Table 3:** Internal consistency reliability of the ‘PCP’s commitment to the vaccination approach’ score in the six vaccine-preventable diseases and overall (*N* = 1,069)

Vaccine-preventable disease	N	Cronbach’s alpha
Measles	209	0.87
Pertussis	199	0.84
Pneumococcus infection	193	0.87
Seasonal influenza	196	0.84
HPV infection	180	0.85
Tetanus	73	0.80
*Overall*	*1050*	*0.86*

*HPV,* human papillomavirus

The results of the construct validity of DIVA thematic scores according to the Delphi panel’s hypotheses are detailed in Table 4. In 30 out of 36 comparisons, the rank obtained by descriptive analyses was identical or adjacent to the rank given by the Delphi panel. Some inconsistencies were observed for pertussis, seasonal influenza and tetanus diseases.

**Table 4:** Comparison of the ranks of DIVA scores given by descriptive analyses of thematic scores and those given by the Delphi panel’s propositions (in parenthesis) – *N* = 1,069

DIVA thematic score	Vaccine-preventable diseases					
	Measles	Pertussis	Pneumococcus infection	Seasonal influenza	HPV infection	Tetanus
Disease characteristics and expected benefits	1 (2)	1 (2)	**1 (1)**	1 (4)	**1 (1)**	**1 (1)**
Properties of the vaccine	2 (1)	2 (3)	2 (3)	3 (1)	**2 (2)**	**2 (2)**
Information about vaccination	**4 (4)**	6 (5)	5 (4)	6 (5)	**5 (5)**	6 (3)
Practical and organisational aspects	**6 (6)**	3 (6)	4 (5)	5 (6)	3 (4)	3 (5)
Adaptation to the patient’s profile	**5 (5)**	5 (4)	**2 (2)**	2 (1)	4 (3)	**4 (4)**
PCP’s individual experience	3 (2)	4 (1)	**6 (6)**	**3 (3)**	**6 (6)**	5 (6)

In bold, identical rank between those given by Delphi panel and descriptive analyses

Difference between rank obtained by descriptive analyses and rank given by Delphi panel =1; =2; =3

## Discussion

In this study, the DIVA questionnaire confirmed its reliability and validity in a large sample of PCPs and across a variety of VPDs. The quality of completion of the DIVA questionnaire was very good. A few missing data were observed for items. The analyses on the hypothetical conceptual framework of the DIVA questionnaire led to some minor adjustments: one item removed and a few changes in three item labels. The Rasch model validated the commitment domain and its six items as a measure of the level of commitment of PCPs in the vaccination against a specific disease [20].

The final 55-item DIVA questionnaire includes the six original thematic domains, covering the concepts measuring a large set of factors that influence PCPs’ attitudes and beliefs toward vaccination including: disease characteristics, vaccine properties, scientific and non-scientific communication, practical and organizational aspects, the patient’s profile and the PCP’s individual experience. The DIVA questionnaire also includes a specific domain assessing the commitment of the PCP to vaccination against the specific disease of interest.

To our knowledge, our study was the first to present simultaneously all the six thematic domains plus a commitment domain in a measurement approach applicable to a large variety of VPDs.

All DIVA thematic domains were identified in the literature, and several studies have explored specific determinants in specific diseases [24]. The most frequently studied domain was related to the ‘Adaptation to the patient’s profile’ domain, which showed the lowest mean score across our study sample. A survey was conducted among pediatricians and family medicine physicians in 2010, before the quadrivalent HPV vaccine (HPV4) was recommended for 11- to 12-year-old boys [25]. The most commonly reported barrier to HPV4 was financing (out-of-pocket cost and lack of adequate reimbursement for vaccine). The second barrier was related to parents’ attitudes, not thinking that HPV4 was necessary for their sons [25]. In 2008, a French survey with 1,000 family physicians showed that physicians who did not think that HPV vaccine had a negative effect on the image of sexuality were more in favour of vaccination [26].

Previous studies on PCPs’ attitudes and practices in vaccination showed that French PCPs’ concerns were focused on the safety of the vaccine [10, 12, 14, 27]. This is partially consistent with our finding, as the ‘Disease characteristics and expected benefits’ domain showed high scores across all studied diseases. However, our study showed that ‘Properties of the vaccine’ domain was not the major concern. Rather, we found that the ‘Information about vaccination’ and the ‘Practical and organizational aspects’ domains constituted the main barriers to vaccination for PCPs. Other studies confirmed that the lack of information on vaccination and the recommendations for vaccination might impact vaccine uptake rates in the general public [10, 12, 14, 27]. The level of acceptance of vaccination by patients was shown to be also an important factor that encouraged PCPs to vaccinate [28–31]. To summarize, it seems that PCPs are fully aware of the advantages of vaccination, generally confident in the vaccines properties, but face difficulties when communicating to patients and addressing practical organization of vaccination, which negatively impact their commitment to vaccination.

In this study, six VPDs were chosen according to their level of acceptance: the vaccines against measles, pertussis, pneumococcus infection and tetanus (compulsory in France) diseases have not been subject to much debate; the vaccines against the seasonal influenza and HPV infection diseases have created controversies [4, 32]. The expected barriers toward vaccination for each of the diseases were assessed with a Delphi Panel approach, and were generally confirmed by our findings in the surveyed population of PCPs. However, a couple of inconsistencies appeared between the rank of DIVA scores given by descriptive analyses and those given by Delphi panel in the pertussis, seasonal influenza and tetanus diseases. Indeed, PCPs seemed less convinced than panellists that the disease characteristics of the seasonal influenza and the expected benefits of vaccination were sufficient to engage them in vaccination. This could have been influenced by the debate in the A/H1N1 outbreak in 2009 [27]. The observed discrepancies between panellists and surveyed PCPs about pertussis and tetanus vaccination are more compelling and need further exploration.

In addition to the assessment of the determinants of PCPs’ attitudes toward vaccination, the DIVA questionnaire specifically measures their commitment toward vaccination against each of the six selected VPDs. The ‘PCP’s commitment to the vaccination approach’ domain had very good internal consistency reliability overall and in the six VPDs, showing its accuracy in measuring the commitment of a PCP to vaccination. In addition, it was validated by a Rasch model, encouraging future researchers to use the ‘PCP’s commitment to the vaccination approach’ as a stand-alone tool either as an outcome measure of intervention programmes encouraging vaccination, or as an explanatory factor in public health research.

We can assume that our survey has not reached PCPs who would have strong beliefs against vaccination. However, the issue is not to convince a minority of opponents about the relevance of vaccination for public health. Rather, the variations observed across VPDs clearly show that the commitment to vaccine is explained by well-identified factors. The DIVA questionnaire is the first standardized tool examining the barriers to vaccination that specific diseases may face and informing where efforts should be concentrated in order to increase adoption of positive attitudes toward vaccination by PCPs.

## Conclusions

The DIVA questionnaire was found to be reliable and valid, allowing PCPs’ attitudes and beliefs toward vaccination to be comprehensively assessed.

In a long-term perspective, the DIVA questionnaire could help develop fine-tuned, efficient interventions to overcome the barriers that prevent PCPs from engaging in vaccination. Ultimately, these interventions will improve vaccine coverage rates in the general population in France.

Furthermore, the findings on the ‘PCP’s commitment to the vaccination approach’ domain of the questionnaire suggest the use of this domain as a stand-alone questionnaire.

## Additional file

Additional file 1: Appendix A. The ‘Determinant of Intentions to Vaccinate’ (DIVA^©^) questionnaire. (PDF 151 kb)

## Abbreviations

CNIL: Commission Nationale de l'Informatique et des Libertés
DIVA: Determinants of Intentions to Vaccinate
HPV: Human papillomavirus
HPV4: Quadrivalent human papillomavirus
MMR: Measles, mumps and rubella
PCA: Principal component analysis
PCP: Primary care physician
RSM: Rating Scale Model
SFMG: Société Française de Médecine Générale
VPD: Vaccine-preventable disease
