# Erratum to: Scoring and psychometric validation of the ‘Determinants of Intentions to Vaccinate’ (DIVA^©^) questionnaire

**DOI:** 10.1186/s12875-016-0567-z

**Published:** 2016-12-19

**Authors:** Luc Martinez, Fatoumata Fofana, François Raineri, Pascale Arnould, Khadra Benmedjahed, Guillaume Coindard, François Denis, Didier Duhot, Jean-Luc Gallais, Didier Seyler, Béatrice Tugaut, Benoit Arnould

**Affiliations:** 1 Mapi, Patient-Centered Outcomes, 27, rue de la Villette, 69003 Lyon, France; 2 French Society of General Medicine, Issy-les-Moulineaux, France; 3 Department of General Practice, University Paris-Sud, Le Kremlin-Bicêtre, France; 4 Department of Bacteriology and Virology, University of Limoges, Limoges, France; 5 Department of General Medicine, University Pierre-et-Marie-Curie, Paris, France; 6 Specialist in general medicine, International vaccination centre (2007–2015), Marseille, France; 7 Department of General Medicine, SMBH University of Paris 13, Bobigny, France

## Erratum

The original article [1] contains an error whereby the Competing interest section does not display the appropriate information.

The section should instead read as such:
