# Supplementary material for: Scoring and psychometric validation of the ‘Determinants of Intentions to Vaccinate’ (DIVA©) questionnaire
Source: BMC Fam Pract. 2016 Oct 10;17:143. doi: 10.1186/s12875-016-0539-3 (PMC5057471; doi:10.1186/s12875-016-0539-3)
Supplement: Additional file 1: — Appendix A. The ‘Determinant of Intentions to Vaccinate’ (DIVA©) questionnaire. (PDF 151 kb) [file 12875_2016_539_MOESM1_ESM.pdf]

Determinants of Intentions to Vaccinate

Vaccination against *[the disease]*

Dear General Practitioner,

This questionnaire is designed to improve understanding of the **factors which influence your attitude towards vaccination against *[the disease]***.

Please **tick only one answer per question**.

Please **answer the questions honestly**. The information in this questionnaire will remain strictly **anonymous** and **confidential**.

Thank you in advance for your participation.

DIVA © SFMG Study – Issy-les-Moulineaux – June 2013 – All rights reserved

DISEASE CHARACTERISTICS AND EXPECTED BENEFITS

**Concerning your attitude towards prescribing the vaccine against *[the disease]*:**	Strongly discourages vaccination	Discourages vaccination	Neither encourages nor discourages vaccination	Encourages vaccination	Strongly encourages vaccination
1. is the benefit to the community a factor which…	☐	☐	☐	☐	☐
2. is the benefit to the patient a factor which…	☐	☐	☐	☐	☐
3. is the efficacy of vaccination compared to that of other existing means of **preventing** *[the disease]* a factor which…	☐	☐	☐	☐	☐
4. is the efficacy of vaccination compared to that of the range of **curative** treatments available against *[the disease]* a factor which…	☐	☐	☐	☐	☐
5. is the diagnosis (easy or difficult) of *[the disease]* a factor which…	☐	☐	☐	☐	☐
6. is the prevalence of *[the disease]* a factor which…	☐	☐	☐	☐	☐
7. is the method of transmission of *[the disease]* a factor which…	☐	☐	☐	☐	☐
8. is the degree of exposure of your patients to *[the disease]* a factor which…	☐	☐	☐	☐	☐
9. is the severity of *[the disease]* a factor which…	☐	☐	☐	☐	☐

DIVA © SFMG Study – Issy-les-Moulineaux – June 2013 – All rights reserved

PROPERTIES OF THE VACCINE

**Concerning <u>your attitude towards prescribing</u> the vaccine against *[the disease]*:**	Strongly discourages vaccination	Discourages vaccination	Neither encourages nor discourages vaccination	Encourages vaccination	Strongly encourages vaccination
10. is the <u>risk/benefit ratio of the vaccine</u> against *[the disease]* a factor which…	☐	☐	☐	☐	☐
11. is the <u>presence of other ingredients</u> in the vaccine against *[the disease]* a factor which…	☐	☐	☐	☐	☐
12. are the <u>strains</u> covered by the vaccine against *[the disease]* a factor which…	☐	☐	☐	☐	☐
13. is the <u>duration of immunisation</u> of the vaccine against *[the disease]* a factor which…	☐	☐	☐	☐	☐
14. is the <u>efficacy of the vaccine</u> against *[the disease]* a factor which…	☐	☐	☐	☐	☐
15. is the level of <u>risk of inducing</u> *[the disease]* via the vaccine a factor which…	☐	☐	☐	☐	☐
16. is the <u>safety of the vaccine</u> against *[the disease]* a factor which…	☐	☐	☐	☐	☐
17. is the <u>method of administration of the vaccine</u> against *[the disease]* a factor which…	☐	☐	☐	☐	☐
18. is the existence - or not - of <u>combined vaccines</u> against *[the disease]* a factor which…	☐	☐	☐	☐	☐
19. is the <u>availability</u> of the vaccine against *[the disease]* a factor which…	☐	☐	☐	☐	☐

DIVA © SFMG Study – Issy-les-Moulineaux – June 2013 – All rights reserved

INFORMATION ABOUT THE VACCINATION

**Concerning <u>your attitude towards prescribing</u> the vaccine against *[the disease]*:**	Strongly discourages vaccination	Discourages vaccination	Neither encourages nor discourages vaccination	Encourages vaccination	Strongly encourages vaccination
20. is the current vaccination schedule a factor which…	☐	☐	☐	☐	☐
21. is the availability of documentation for your patients at the surgery a factor which…	☐	☐	☐	☐	☐
22. is the content of the scientific literature available to you a factor which…	☐	☐	☐	☐	☐
23. is the communication from NHS England or your CCG on this subject a factor which…	☐	☐	☐	☐	☐
24. is the communication from health institutions (Public Health England, NICE, CQC, etc.) on this subject a factor which…	☐	☐	☐	☐	☐
25. is the communication from the Department of Health on this subject a factor which…	☐	☐	☐	☐	☐
26. is the content of the information supplied by the pharmaceutical companies a factor which…	☐	☐	☐	☐	☐
27. is the content of the information communicated by the media a factor which…	☐	☐	☐	☐	☐

PRACTICAL AND ORGANISATIONAL ASPECTS

**Concerning <u>your attitude towards prescribing</u> the vaccine against [the disease]:**	Strongly discourages vaccination	Discourages vaccination	Neither encourages nor discourages vaccination	Encourages vaccination	Strongly encourages vaccination
28. is the GP's role in the vaccination process a factor which…	☐	☐	☐	☐	☐
29. are the alert settings in your electronic patient records a factor which…	☐	☐	☐	☐	☐
30. are reminder messages from specialists or from occupational healthcare professionals a factor which…	☐	☐	☐	☐	☐
31. is the time needed to follow up, talk about and explain this vaccination during a consultation a factor which…	☐	☐	☐	☐	☐
32. is the financial cost to the community a factor which…	☐	☐	☐	☐	☐
33. is the patient-doctor relationship a factor which…	☐	☐	☐	☐	☐

DIVA © SFMG Study – Issy-les-Moulineaux – June 2013 – All rights reserved

ADAPTATION TO THE PATIENT'S PROFILE

**Concerning <u>your attitude towards prescribing</u> the vaccine against *[the disease]*:**	Strongly discourages vaccination	Discourages vaccination	Neither encourages nor discourages vaccination	Encourages vaccination	Strongly encourages vaccination
34. is the <u>availability of information on whether the patient has already been vaccinated against</u> *[the disease]* a factor which…	☐	☐	☐	☐	☐
35. are the <u>patient's travel plans</u> a factor which…	☐	☐	☐	☐	☐
36. is the fact that a patient belongs to a <u>risk group</u> with regards to *[the disease]* a factor which…	☐	☐	☐	☐	☐
37. is the consultation of a <u>new patient</u> a factor which…	☐	☐	☐	☐	☐
38. is a consultation for an <u>acute illness</u> a factor which…	☐	☐	☐	☐	☐
39. is a consultation for a <u>chronic illness</u> a factor which…	☐	☐	☐	☐	☐
40. is the <u>multiple morbidity of a patient</u> a factor which…	☐	☐	☐	☐	☐
41. are <u>patients' knowledge</u> and <u>preconceived ideas</u> about the vaccination against *[the disease]* a factor which…	☐	☐	☐	☐	☐
42. is the <u>link between *[the disease]* and taboos,</u> beliefs or behavioural norms a factor which…	☐	☐	☐	☐	☐
43. is the <u>level of acceptance by your patients</u> of the vaccination against *[the disease]* a factor which…	☐	☐	☐	☐	☐
44. is the <u>cost to be covered by your patients</u> for vaccination against [*the disease*] a factor which…	☐	☐	☐	☐	☐

DIVA © SFMG Study – Issy-les-Moulineaux – June 2013 – All rights reserved

GENERAL PRACTITIONER'S INDIVIDUAL EXPERIENCE

**Concerning <u>your attitude towards prescribing</u> the vaccine against *[the disease]*:**	Strongly discourages vaccination	Discourages vaccination	Neither encourages nor discourages vaccination	Encourages vaccination	Strongly encourages vaccination
45. are the <u>characteristics of the population</u> you treat (age, sex, geographic location, etc.) a factor which…	☐	☐	☐	☐	☐
46. is the degree of <u>difficulty in identifying the target population</u> for vaccination against *[the disease]* a factor which…	☐	☐	☐	☐	☐
47. are the <u>cases of *[the disease]* encountered within the scope of your professional experience</u> a factor which…	☐	☐	☐	☐	☐
48. is your <u>experience</u> of the <u>vaccination</u> against *[the disease]* <u>on a personal level</u> (yourself or your family) a factor which…	☐	☐	☐	☐	☐
49. is your <u>experience of *[the disease]* on a personal level</u> (yourself or your family) a factor which…	☐	☐	☐	☐	☐

GENERAL PRACTITIONER'S COMMITMENT TO THE VACCINATION APPROACH

**<u>During a consultation with a patient at risk</u> of *[the disease]*,**	Totally disagree	Somewhat disagree	Somewhat agree	Totally agree
50. <u>I raise the subject</u> of vaccination against *[the disease]*	☐	☐	☐	☐
51. <u>I prescribe</u> vaccination against *[the disease]*	☐	☐	☐	☐
52. <u>I insist</u> on vaccination against *[the disease]* if the <u>patient is reluctant</u>	☐	☐	☐	☐
53. <u>I make sure</u> that my <u>prescription</u> for vaccination against *[the disease]* has been properly <u>followed</u>	☐	☐	☐	☐

**In general,**	Totally disagree	Somewhat disagree	Somewhat agree	Totally agree
54. vaccination against *[the disease]* is <u>a subject that interests me</u>	☐	☐	☐	☐
55. my <u>attitude towards prescribing</u> the vaccine against *[the disease]* is <u>in agreement with my beliefs</u>	☐	☐	☐	☐

Thank you for your time.

DIVA © SFMG Study – Issy-les-Moulineaux – June 2013 – All rights reserved
